# Role of the epithelium in human papillomavirus and human immunodeficiency virus infections in the female genital tract

**DOI:** 10.3389/frph.2024.1408198

**Published:** 2024-05-02

**Authors:** Sengeziwe Sibeko, Micheline Sanderson, Sizulu Moyo, Matthys H. Botha

**Affiliations:** ^1^ Public Health, Societies and Belonging Division, Human Sciences Research Council, Durban, South Africa; ^2^ Division of Anatomical Pathology, Department of Pathology, Stellenbosch University, Cape Town, South Africa; ^3^ Department of Obstetrics and Gynaecology, Stellenbosch University, Cape Town, South Africa

**Keywords:** HPV, HIV-1, epithelium, barrier, disruption, female genital tract

## Abstract

**Background:**

Two-thirds of people living with human immunodeficiency virus type 1 (HIV-1) infection reside in Sub-Saharan Africa, where there are the highest prevalence and incidence rates of human papillomavirus (HPV) infection. Both infections are sexually transmitted and enter the body via the epithelium. This review describes the extent of involvement of the epithelium in each infection in the female genital tract.

**Methods:**

A narrative review was conducted on the role of the epithelium in HPV and HIV-1 infections.

**Results:**

An intact epithelial barrier is the predominant form of protection against viral entry and infection, including from HIV-1 and HPV. HPV is an intraepithelial pathogen, and thus, its growth and amplification, which are dependent on squamous cell differentiation, occur in the epithelium. It gains entry to the basal cells of the stratified squamous epithelium via micro-abrasions or other epithelial injuries that expose the basement membrane. HIV-1, conversely, passes through the epithelium to infect subepithelial tissues. Following deposition of the HIV-1-containing inoculum into the lumen, the virus enters the mucosa through breaks in the epithelial barrier within hours of infection. Further, HIV-1 penetrates the epithelium via various mechanisms, including paracellular passage or across epithelial cells through transcytosis. The capture of the virus from the mucosal surface by intraepithelial and/or subepithelial target cells has also been documented.

**Conclusions:**

Epithelial disruption is the major pathogenetic pathway in HIV-1 and HPV infections. Therefore, biochemical compounds that strengthen the epithelial barrier must be prioritized to prevent these infections.

## 1 Introduction

Mucosal tissues in the human female genital tract are prone to invasion by various pathogens including Human Immunodeficiency Virus type 1 (HIV-1) and Human Papillomavirus (HPV). Further, they are primary sites for gynecological cancers and other sexually transmitted infections (1). HPV and HIV-1 infect or traverse the epithelium, respectively. Here we discuss the connection to and extent of involvement of the epithelium in HPV and HIV-1 infections.

## 2 Epidemiology of HPV and HIV-1

Worldwide, HPV, a highly transmissible sexually transmitted virus, is responsible for 5% of all cancers (2), accounting for slightly more than 30% of infection-linked cancers (3). Of the various body sites that can be affected by HPV infection and associated cancers, cervical cancers account for over 90% of HPV-related cancers in women. The prevalence of cervical HPV is highest in Sub-Saharan Africa (SSA), followed by Latin America and the Caribbean, eastern Europe, and South-East Asia at 24%, 16%, 14% and 14%, respectively (2).

To date, at least 200 HPV types have been identified and grouped within 5 genera (4–6). The 65 alpha papillomaviruses known to target mucosal membranes can be divided into high-risk (hr) and low-risk (lr) groups based on their ability of oncogenic transformation of cells (5–8). Clinically, persistent hrHPV infections are associated with both invasive cancer of the cervix and cervical intraepithelial neoplasia (CIN), a precursor lesion for cervical carcinoma. Persistent infection with hrHPV has been linked to more than 99% of cervical cancer cases, whereas 90% of CIN cases occur in HPV-positive patients (9). HPV16 and HPV18 are associated with high grade intraepithelial lesions (HSIL), encompassing CIN2 and CIN3 lesions, and invasive cancer progression (10). HPV16 infection is closely associated with both intraepithelial lesions and invasive squamous neoplasia, as well as the occasional cervical glandular neoplasia (11). HPV18 on the other hand is associated with non-squamous cervical neoplasms and it has been detected in about 9% of HPV infections (12). However, more than 90% of any cervical HPV infection will regress (13). In the absence of regression, HPV infection can persist for decades, becoming a major risk factor for later neoplastic transformation of cervical epithelium and progression from intraepithelial lesions to invasive carcinomas (14).

HIV-1, another sexually transmitted pathogen, is a cause of acquired immunodeficiency syndrome (AIDS), a chronic multisystem and potentially life-threatening condition that compromises immunity, thereby interfering with the body's ability to fight infection and disease. Globally, an estimated 1.3 million people were newly infected with HIV-1 in 2022 (15). Furthermore, over the same period, 630 00 people died from AIDS (15). Globally, 39 million people were living with HIV (PLHIV) (15). While the incidence of HIV infection has been declining, it has not been declining at high rates in women and girls. Forty six percent of all new HIV infections in 2022 were in women and girls, and fifty three percent of all PLHIV were women and girls (15).

There is a high HPV prevalence among women living with HIV (WLHV). Stelzle et al., found that globally WLHV have six times the risk to develop cervical cancer than their uninfected counterparts (16). Mbulawa et al, in a study conducted in South Africa, showed that the prevalence of HPV among HIV infected women was 74.0% compared to 36.7% in HIV uninfected women, a difference that was statistically significant (17). In a meta-analysis by Clifford et al.*,* HPV prevalence in HIV infected women with normal cytology was 36.3% for any HPV whereas for infection with multiple types of HPV, it was 12% (18). Further, out of a total of 5,578, HPV16 was the most common type of HPV found in HIV infected women with either atypical squamous cells of undetermined significance (ASCUS) or low-grade squamous intraepithelial lesion (LSIL) (18). HPV16 was also found in 295 women with high-grade intraepithelial lesion (HSIL) (18). Lastly, together with Kaposi's sarcoma and B-cell non-Hodgkin lymphoma, invasive cervical cancer have been considered to be the three AIDS-defining cancers (ADCs) PLHIV (19). However, recently, additional cancers in various sites such as head and neck, anogenital, lung, and skin have been described and shown to be associated with HIV-1 infection (19).

## 3 Structure and role of the female genital tract epithelium in HPV and HIV-1 infections

The mucosa forms the innermost layer of the wall of luminal structures in the body, including the female genital tract (FGT). The mucosa consists of (i) the epithelium, as the topmost layer, (ii) an underlying loose connective tissue layer, (iii) a thin layer of smooth muscle called the lamina propria, and (iv) the muscularis mucosa. An intact epithelial barrier forms the predominant defense site against viral invasion and infection (20). A compromised epithelial barrier may enhance viral exposure to underlying target cells (20).

**Figure 1:**
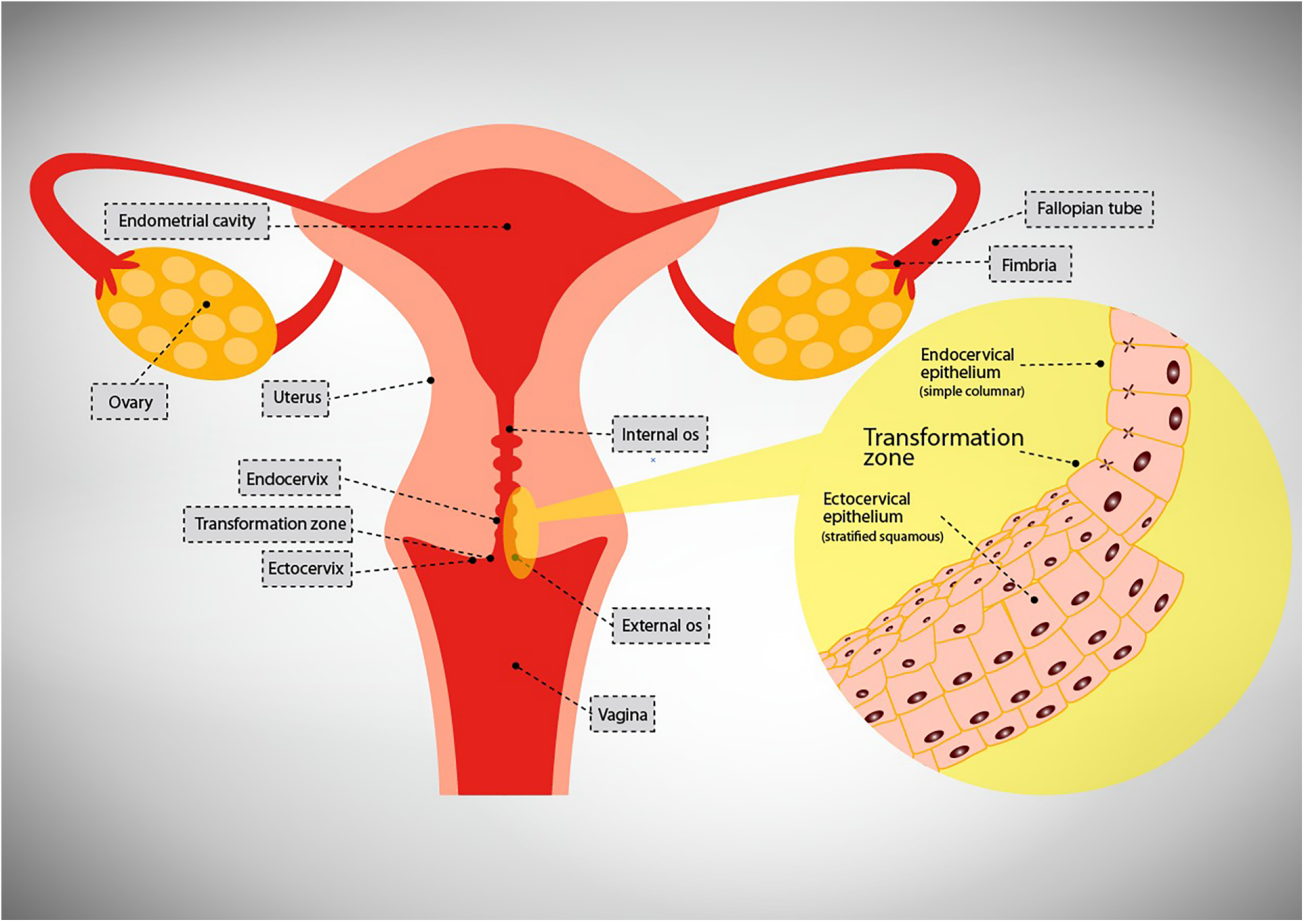
Depiction of the compartments of the female genital tract.

The female genital tract (FGT) is one of the organ systems of the body that connect the internal to the external environments and as such it is lined by the epithelium. The upper genital tract (UGT) comprises the uterus, fallopian tubes, ovaries, and endocervix while the lower genital tract (LGT) comprises the ectocervix, vagina and vulva (21) (Refer Figure 1)*.* A single layer of simple columnar cells lines the UGT while multilayered pluristratified squamous cells line the lower genital tract (LGT) i.e., ectocervix and vagina.

The human vagina has a large surface area, ranging from 65.8 to 107.1 cm^2^ with a mean of 87.5 cm^2^ (22) and is the first mucosal surface in the LGT to come into contact with sexually transmitted pathogens (23), and is usually successful in impeding invasive infections. In contrast, albeit having a smaller surface area of approximately 4 cm in length and 3 cm in diameter, the endocervix (24), is a site frequently infiltrated by numerous sexually transmitted pathogens including HPV and HIV-1.

It is estimated that 30%–40% of all new HIV-1 infections in women occur following heterosexual intercourse via mucosal surfaces in the FGT (25), while an estimated sixty percent of HPV infections in women occur in the uterus (26). On entry into the FGT microorganisms such as HIV-1 and HPV interact with epithelial cells, and the initial innate immune responses are usually adequate to avert the infection at this stage. However, if they are not, then the epithelial signaling mechanisms of the host are activated and the infection is established (27).

### 3.1 Mechanisms to limit access to the basement membrane and subepithelial tissues

Mechanisms adopted by epithelial cells to hinder establishment of infection include their architecture, which includes tight intercellular junctions; production of mucus; secretion of innate immune mediators and changes in thickness of the epithelial lining secondary to fluctuations in hormone levels (28).

Cells of the simple epithelium are polarized having apical and basolateral domains. The apical domain faces the lumen while the basolateral domain faces the serosal aspect (29). This layer's high degree of impermeability to incoming pathogens is mostly due to the function of tight junctions. Contrary to the simple columnar epithelial layer, the pluristratified epithelial layer does not have a polarized plasma membrane or tight junctions. Owing to this lack of tight junctions, extracellular molecules or other cell types are free to diffuse between cells, a process referred to as paracellular passage (30).

The endocervical surface is highly convoluted, containing goblet cells that are continually producing thick mucoid secretions that are able to trap pathogens so that they are not transmitted across the epithelium (31). These secretions not only house an array of immunomodulatory proteins but also house an array of antimicrobial molecules which protect against external environmental challenges (28). Further, antibodies that are capable of neutralising the virus coat the columnar epithelium of the endocervix, thus enhancing its protective role (32). By secreting antimicrobial factors, epithelial cells are able to eliminate potential pathogens (33). Antimicrobial peptides form an extra protective layer of the endocervix and exert their effect by disrupting microbial membranes and the metabolic processes of microbes (33).

One of the distinctive roles of the epithelium includes the detection of harmful stimuli through pattern recognition receptors (PRRs) and then to relay this information to the adaptive immune system through secretion and up-regulation of various cytokines and chemokines (34). These cytokines and chemokines have immunomodulatory functions and their secretion may be constitutive or induced by stimuli such as the virus itself or the presence of inflammation (35). The functions of the epithelium that are regulated by cytokines and chemokines include its ability to first and foremost recruit and then to activate and regulate immune cells of both the innate and adaptive immune systems to the site of injury (36).

Lastly, the distinct anatomical regions of the FGT undergo epithelial morphological changes related to the menstrual cycle fluctuations in levels of oestradiol and progesterone (37). Estrogen is responsible for proliferation, maturation, and desquamation of all layers of the multilayered squamous epithelium which enhances the risk of viral invasion (37). On the other hand, progesterone causes thickening of intermediate layers and thereby protecting against viral entry (38).

## 4 Epithelial involvement by HPV and HIV-1 in the FGT

### 4.1 HPV in the epithelium

HPVs are said to be wholly intraepithelial pathogens (39, 40). Infection with HPV and its growth are dependent upon the machinery of keratinocyte differentiation (40). HPV infects basal cells of stratified squamous epithelium which it does through micro-abrasions which may occur following trauma that results in parts of the basement membrane being exposed (41). However, its incubation period varies in duration, ranging from a few weeks (3–4 weeks) to months or even years (42). It is postulated that this duration is dependent on the received amount of virus (42).

Currently, there is consensus that heparan sulfate proteoglycans (HSPGs) are the critical primary attachment factors for epithelial cells following infection with some HPV types (41). The virus binds to its target, the basement membrane or the surface of basal layer cells via receptors located on the surface of those structures (43, 44). Cell surface binding is followed by internalisation of the virus, the initial and slow phase of infection. The internalisation process of HPV usually occurs about 2–4 h after cell surface binding (41). The endocytic pathways and intracellular trafficking of the viral capsid at play during the process of internalization have been extensively studied, however, consensus is limited (41). This is partly because different genotypes use various pathways. Nonetheless, regardless of genotype, the process of internalization occurs slowly in contrast to most other virus types, where internalisation occurs rapidly within minutes of cell surface binding (41).

After accessing the cellular internal structures, the process of transcription occurs following entrance of the episomal genome into the nucleus (43). Viral replication occurs alongside that of the viral target cells i.e., the basilar cell. Subsequently viral genomes that have been integrated into host chromosomes are then split to new daughter cells during mitosis (43). Viral genome copies are increased and particles are formed when virus-infected basal cells develop into keratinocytes (41). As amplification occurs, the initial round of viral particles are amplified to approximately 100 nuclear episomes per cell (42). While there is low viral gene expression in the growing upper layers of the pleuristratified epithelium, the virus persists in an episomal maintenance phase for a certain amount of time. Thousands of viruses are present in the keratinocytes by the time they desquamate from the outermost layers of the epithelium. After that, desquamated cells are prepared to infect the next host (40).

### 4.2 HIV-1 in the epithelium

HIV-1 passes through the epithelium to infect subepithelial tissues. HIV-1 passes through the epithelium to infect subepithelial tissues. A number of receptors and coreceptors have been elucidated that interact with viral envelope protein gp120, including C-X-C chemokine receptor type 4 (CXCR4), C-C chemokine receptor type 5 (CCR5), galactosylceramide (GalCer), heparan sulfate proteoglycans (HSPGs), and mannose receptors, as well as integrins, and Toll Like Receptors 2/4 on the mucosal surface (45–51).

The virus traverses the epithelium quickly following exposure to the viral containing inoculum in the lumen (52). Transmission of HIV-1 through mucosal epithelium is the first step that is crucial to HIV-1 gaining access to the various systems of the body and thereby causing AIDS (53). While recent studies show that HIV-1 may interact with the epithelium, the main function of the epithelium is to aid the virus in passing through to the submucosa and the systemic compartments (54).

According to Haase et al., following mucosal exposure to high doses of simian immunodeficiency virus (SIV) in non-human primates, the virus passes through the epithelium to reach the subepithelial tissues within a few hours of inoculum deposition (54). Once in the subepithelial tissues, the founder population which is the virus with infected resting CD4+ *T* cells, is formed (54). The founder population subsequently enlarges in size at this site, a process that is called local mucosal expansion. This phase is followed by dissemination of infection to the regional draining lymph nodes. Ultimately the infection reaches the systemic compartment where it causes widespread infection following a process of self-regenerating infection in the secondary lymph nodes.

HIV-1 entry may occur via any FGT subcompartment with distinct epithelial architecture (55). HIV-1 can penetrate the epithelium via four different mechanisms at least. As the initial step, the presence of underlying microlacerations or microabrasions is important. These mechanisms include paracellular passage, trancytosis, capture of virus by intraepithelial or subepithelial cells and via infection of the epithelial cells (56, 57). Paracellular passage occurs following epithelial disruption. Transcytosis, a form of transcellular passage, is the process whereby virions are transported across the epithelium by vesicular or endosomal machinery of epithelial cells (58). Even though it has been described for squamous cells of the pseudostratified epithelium, it occurs mainly in columnar cells as are found in the endocervical canal (59). The intrinsic characteristics of the epithelium usually determine which passage strategy HIV-1 is going to employ as does the type of the epithelium. No consensus has been reached as to which mechanism of HIV-1 passage across the epithelium is predominant.

## 5 Discussion

Mucosal tissue serves as a protective barrier in the FGT and is the main target site for gynaecological cancers and infections by a spectrum of sexually transmitted pathogens, including HPV and HIV-1 (18). Consequently, a compromised mucosal barrier, and particularly the epithelium, increases the risk of acquiring these latter two viral infections. Recognised factors contributing to a broken or fragile epithelium include cervical ectopy (60), fluctuations in levels of reproductive hormones which may lead to a thin epithelium (38), high-risk sexual behaviour such as unprotected sex and early coitarche (61), co-presence of other sexually transmitted infections (62, 63), altered microbiome (64, 65) and inflammation in HIV-1 infection (66). In addition, there is an association between HPV and HIV-1, with the one worsening the other's pathogenesis (67, 68). Therefore, medical interventions directed at addressing these underlying mechanisms to acquiring HPV and HIV-1, could potentially prevent, halt or even treat these infections.

Primary prevention against HPV infection is by the HPV vaccine. The HPV vaccine first licensed in 2006 has been adopted by many countries (69). In 2022, an estimated 125 countries (64%) had adopted HPV vaccination in their national immunization programmes for girls (69). While this programme has largely been successful in protecting against HPV and curbing cervical neoplasia and invasive cervical cancer in many developing countries, there have, however, been structural and programmatic issues that have hindered it from being widely available and thus wholly successful (70). On the other hand, to date, there is no preventive vaccine against HIV-1.

With regards to FGT infection, the earliest phases of infection at the point of entry, where there are the most host immune strengths and most viral weaknesses, offer the best chances for effective interventions to prevent sexual viral transmission (54). As a compromised epithelium is the primary route for infection, targeting this area with products to mitigate infection is warranted. This is particularly the case as, by and large, there is understanding in the literature of (i) the baseline innate epithelial status as it pertains to secretion of infection-modulating molecules, (ii) endocrine regulation of these molecules over different phases of the menstrual cycle, and (iii) the earliest innate epithelial responses following exposure to viruses that achieve these aims. Thus, such pathogenetic pathways could be exploited to develop products that enhance the effectiveness of these immune responses.

Particularly, there are multipurpose prevention technologies (MPTs) which are products that aim to simultaneously prevent at least two sexual and reproductive health conditions inclusive of unintended pregnancy, HIV and other STIs including HPV (71). According to Boonstra, “There is a compelling need for new technologies that protect sexually active women against multiple sexual and reproductive health risks, especially in countries heavily burdened by HIV, HPV and by maternal and infant mortality” (72). To date, MPTs have come in various tested preparations and delivery methods, including intravaginal rings, vaginal and rectal gels, vaginal inserts and films, systemic delivery implants, subdermal microarray patches, and oral tablets containing contraceptives, anti-HIV and/or other STI prevention drugs (73). Particularly, Population Council looked at PC-1005 gel, also known as MZC gel, comprising of three key ingredients that provide broad-spectrum antiviral activity against HIV, HSV and HPV (74). In a Phase I clinical trial, this product was found to be promising with high acceptability levels (75). However, it is yet to be tested in higher clinical trial stages. Recently, the use of monoclonal antibodies (mAbs) as MPTs in the prevention and treatment of HIV-1 and other STIs have come to fore and several research studies are underway. Some of these have gone to clinical trial stages and they include MABgel and VRC01/MB66 topical vaginal film against HIV, amongst others, and 2C (HDIT101), E317 (UB-621) and HSV8 against HSV (76). Dohadwala et al., in their paper concluded that it is ideal to develop mAbs that will target multiple STIs concurrently (76).

Some other interesting studies have looked at the combination of antivirals as targets for both HIV and HPV. Specifically, Hampson et al., studied a combination of lopinavir and ritonavir, oral drugs used in the treatment of HIV, inserted intravaginally, as a potential treatment for CIN (77). Indeed, they were able to show at 12 weeks that there was an estimated 80% regression in cytology results from HSIL to either low grade or no dysplasia, a finding that was confirmed on histology (77). Further, about 50% had no detectable HPV. As the authors pointed out, these findings highlight the potential of the combination of anti-HIV drugs as a self-administered treatment for cervical lesions associated with HPV infection. What would be interesting is the use of these drugs as prevention of both these conditions.

## 6 Strengths and limitations

In some regions of the world, such as Sub-Saharan Africa, there are high prevalence and incidence rates of both HPV and HIV-1 infections, and hence, they are overlapping public health enigmas, and eradicating one might impact the prevalence and impact of the other. To our knowledge, no side-by-side review has been done on the extent of the involvement of the epithelium in these two infections. This review therefore highlights areas of overlap and divergence between HPV and HIV-1 infections with regards to their involvement of the epithelium in their pathogenesis. The review ends by suggesting biomedical means of targeting both infections simultaneously. Conversely, the strength of this paper as a narrative review is that it remains broad, providing a general background on the topic. It could serve as a stepping stone to doing a more comprehensive review, such as a scoping review. Lastly, it does not necessarily shed light on guidelines for managing these infections.

## 7 Conclusion

Both FGT HPV and HIV-1 have varying degrees of epithelial involvement in their pathogenesis. HPV infection being exclusively intraepithelial and HIV-1 infection a systemic disease but requiring to traverse the epithelium to establish systemic infection. Breaches in the epithelium from various causes in the FGT enhance infection with both of them. We, therefore, need to focus our research on studying different formulations that can target both HIV and HPV simultaneously, and to prioritize research on different formulations that enhance the different aspects of the immune system to enforce their protective pathways.
